# A method to study electronic transport properties of molecular junction: one-dimension transmission combined with three-dimension correction approximation (OTCTCA)

**DOI:** 10.1038/srep21946

**Published:** 2016-02-25

**Authors:** Ran Liu, Chuan-Kui Wang, Zong-Liang Li

**Affiliations:** 1 School of Physics and Electronics, Shandong Normal University, Jinan, 250014, China

## Abstract

Based on the *ab initio* calculation, a method of one-dimension transmission combined with three-dimension correction approximation **(OTCTCA)** is developed to investigate electron-transport properties of molecular junctions. The method considers that the functional molecule provides a spatial distribution of effective potential field for the electronic transport. The electrons are injected from one electrode by bias voltage, then transmit through the potential field around the functional molecule, at last are poured into the other electrode with a specific transmission probability which is calculated from one-dimension Schrödinger equation combined with three-dimension correction. The electron-transport properties of alkane diamines and 4, 4′-bipyridine molecular junctions are studied by applying OTCTCA method. The numerical results show that the conductance obviously exponentially decays with the increase of molecular length. When stretching molecular junctions, steps with a certain width are presented in conductance traces. Especially, in stretching process of 4, 4′-bipyridine molecular junction, if the terminal N atom is broken from flat part of electrode tip and exactly there is a surface Au atom on the tip nearby the N atom, the molecule generally turns to absorb on the surface Au atom, which further results in another lower conductance step in the traces as the experimental probing.

It is noticeable that, due to the rapid progress of single-molecule technologies^1^,^2^,^3^,^4^, various kinds of studies on single-molecules or atomic clusters have been presented recently^5^,^6^,^7^,^8^, and the results of experimental probing of molecular devices are more and more stable and often consist very well with each other although different technologies are employed^8^,^9^. Simultaneously, many theoretical methods have been developed which give deep insights into the electronic transport mechanisms of molecular junctions^10^,^11^,^12^,^13^,^14^,^15^,^16^,^17^,^18^,^19^,^20^,^21^,^22^, such as non-equilibrium electron transport^11^, elastic and inelastic electron transports^12^,^21^, photoinduced charge transport^19^, scattering or tunneling effects^17^,^23^, dimension effect of electrodes^15^, and so on. Based on those theoretical models, the functional properties of single-molecule devices have been studied systematically, including molecular rectifier^24^, molecular switch^8^, molecular transistor^25^, etc. However, there still exist some interesting problems for theoretical studies to deal with which even include the familiar or acknowledged characters of molecular devices. As relatively simple systems, alkane series with different linker groups are representative examples whose electronic transport properties were investigated experimentally many times and the results were almost close to each other^26^,^27^,^28^. It is shown that, in the lower bias voltage regime, their conductance (*G*) decreases exponentially with a decay constant 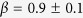 per CH_2_ group^26^,^29^,^30^. The conductance of phenyl chains^31^,^32^ and OPE chains^33^ also shows exponentially decaying with molecular length. It is appreciated that some theoretical groups have successfully studied the length dependence of molecular conductance and given valuable explanation^23^,^34^,^35^,^36^,^37^,^38^. On the base of the studies, we find that the exponential decaying character of length dependence seems still having some difficulties to be simulated theoretically when the same series molecular chains are studied^39^, sometimes even the opposite results to the experiments are given^40^. Additionally, in theoretical studies, the interface configurations perform more influence on the electronic transport properties of molecular junctions than in experimental ones^41^,^42^,^43^. Hence, when molecular junctions are stretched, steps in conductance traces often as a signature of the molecular junction formed in the experiment are seldom simulated out theoretically^44^,^45^. In fact, details of the interface configurations are still difficult to control or even detect in the present experiments^46^,^47^. However, existence of steps in the conductance traces, especially the similar conductance value of steps of different samples, seems to indicate that the conductivities of molecular junctions are insensitive or less sensitive to the factors of interface configuration and electrode distance, which is difficult to be understood for theoretical studies.

As one knows, in the lower bias voltage regime, resonant transmissions contributed by delocalized transmission channels are often very weak. Hence, nonresonant transmissions, which attribute to the quantum scattering and quantum transmission effects of effective potential fields^48^ that are around functional molecules, will be expected to dominate electron-transport properties of molecular junctions. Thus, considering above problems, we develop a method of one-dimension transmission combined with three-dimension correction approximation (OTCTCA) to study the electronic transport properties of molecular junction with lower bias voltages. This method is further used to investigate electron-transport properties of alkane diamines and 4, 4′-bipyridine molecular junctions taken as examples. The results demonstrate that the OTCTCA method not only successfully simulate the length dependence of the molecular conductance, but also reveal the reason that the conductance traces often show steps (even double steps) when molecular junctions were elongated in experimental investigations^49^,^50^,^51^. On the basis of our numerical calculations, we find that the OTCTCA method is very feasible to investigate properties of weak coupling molecular junctions.

## Results

### Molecular length effect

**Figure 1:**
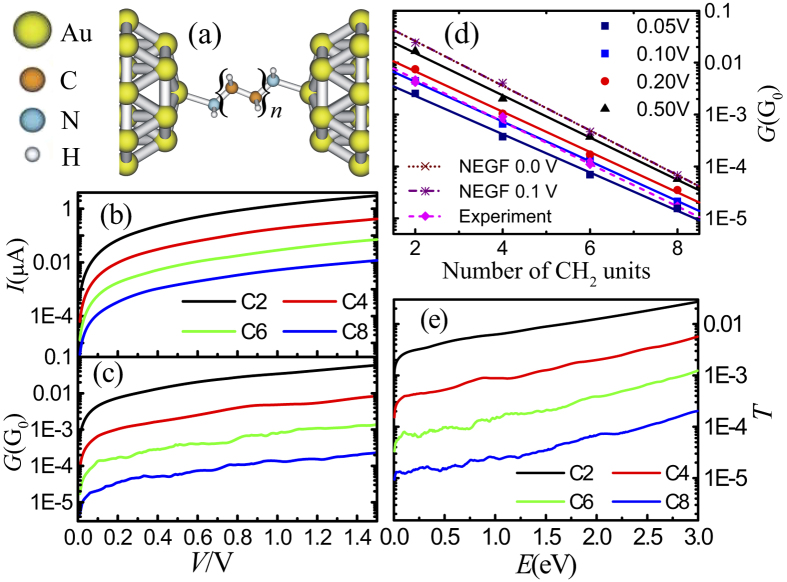
Electronic transport properties of alkane diamines. (**a**) Schematic structures of alkane diamines 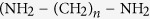, *n* = 2, 4, 6, 8) molecular junctions, (**b**) Current and (**c**) Conductance as functions of bias voltage for the four molecular junctions, (**d**) Conductance of all alkane diamines against the number of 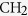 units in the molecular chains, also shown are the linear fits to the data on the semi-logarithm scale. The (magenta) dashed line corresponds to the experiment results^29^,^30^. The results with NEGF method are also included (dotted and dashdotted lines). (**e**) Transmission probabilities as functions of electronic energies for the molecular junctions.

The electronic transport properties of alkane diamines 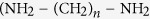, *n* = 2, 4, 6, 8, denoted as C2, C4, C6 and C8, respectively) molecular junctions experimentally probed by different groups^29^,^30^ are firstly investigated by applying OTCTCA method. In order to simulate the influences of gold electrodes on the molecules, considering the screening effects of inner Au layers and the reliability of results, we construct gold-molecule-gold extended molecular systems by sandwiching the molecules between two gold-atom clusters to simulate molecular junctions (Fig. 1(a)) in the calculations. The stretching processes of molecular junctions are simulated by adjusting the distance (*D*) between the two electrodes. For each distance, the molecule and some Au atoms which close to the molecule are relaxed by performing geometric optimization, and the other Au atoms are fixed. Then the electrodes’ distance is elongated by adjusting the fixed Au atoms a little, and the relaxed part of the system that is just optimized is taken as the initial geometry to perform further geometric optimization. By this way, the stretching process is implemented at B3LYP level with Lanl2DZ basis set in Gaussian03 packages^52^.

The numerical results show that, for each gold electrode, one Au atom can be protruded out by the pulling of the functional molecule with elongation of electrodes. The minimum energies occur at *D* = 0.95, 1.21, 1.47 and 1.73 nm for C2, C4, C6 and C8 molecular junctions, which correspond to the equilibrium electrode distances (see Figure S1). When the electrode distances of the four molecular junctions are further elongated to about *D* = 1.06, 1.33, 1.61 and 1.87 nm, the molecular junctions are broken up, and the corresponding broken forces are 0.64, 0.74, 0.78 and 0.77 nN (see Figure S1), respectively, which agree with experimental probes very well^53^,^54^. Just before the molecular junctions being broken, the maximum protruded values of the Au atoms are about 0.091, 0.101, 0.109 and 0.111 nm for C2, C4, C6 and C8 molecular junctions, respectively. After molecular junctions being broken, the protruded Au atoms snap back automatically. Then at equilibrium distances, the effective potential values of grid points of the three-dimension rectangular cubic space that around the molecules are computed. The distance between neighbor grid points is 0.01 nm in directions of *x*, *y* and *z* axes, so it needs about 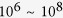 grid points to describe the effective potential for each molecular system. Applying the OTCTCA method with the grid-point potential values, we calculate the electronic transmission for each molecular junction.

Figure 1(b,c) show current and conductance of the four molecular systems. The figures show that both current and conductance increase with bias voltage for all of the four molecular junctions, because the transmission probabilities generally increase with enhancement of the incident energies of electrons in quantum well transmission and quantum barrier tunneling. These characters of transmissions are also represented for the four molecular junctions in Fig. 1(e). In addition, more CH_2_ units in the alkane chains result in lower current, conductance and transmission probability values. The conductance as a function of molecular length expressed by the number of CH_2_ units is plotted in Fig. 1(d) in semi-logarithm scale. From the figure, one can clearly see that the conductance shows exponentially decreasing with increase of the number of CH_2_ units in the molecular chains with 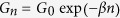, where *n* is the number of CH_2_ units and *β* = 0.850, 0.881, 0.895 and 0.937 for bias voltages *V* = 0.05, 0.10, 0.20 and 0.50 V, respectively. However, when the bias voltage increases up to 1.0 V, the decaying factor is almost unchanged with 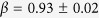, which are in a good agreement with the experiment ones^29^,^30^. The non-equilibrium Green’s function (NEGF) calculations are also performed for those alkane diamine molecular junctions, which show that *β* = 0.991 for bias voltages *V* = 0, 0.10 and 0.20 V (Fig. 1(d)). One can see that our OTCTCA results are only a little smaller than the NEGF ones. It is noticeable that the typical nonresonant transmission character that the conductance increases with the increase of bias voltage^3^,^55^ is not found in the NEGF results. In fact, the exponentially decaying of conductance versus molecular length exactly indicated that the electronic transports of alkane series are as ones expected mainly contributed by nonresonant transmission which results from quantum scattering and quantum transmission effect of effective potential fields that around the molecules. As one knows, resonant transmission is mainly influenced by the factors such as (a) couplings between electronic states of functional molecule and metal electrodes, (b) delocalization of molecular orbitals and (c) whether the delocalized molecular orbitals come into bias window^10^,^11^,^12^. It is obviously that neither of these factors is related to the molecular length. However, the transmission probability of nonresonant transmission is influenced by (a) the number of potential wells and potential barriers, (b) the shape of potential wells and potential barriers, (c) the width and the height of potential barriers and (d) the energy of incident electrons. Thus each repeated unit in the molecule is just as repeated potential wells and potential barriers that will reduce the transmission probability by about equal proportions, which induces the exponentially decaying character of conductance with molecular length for the same series of molecules.

### Step in conductance trance

**Figure 2:**
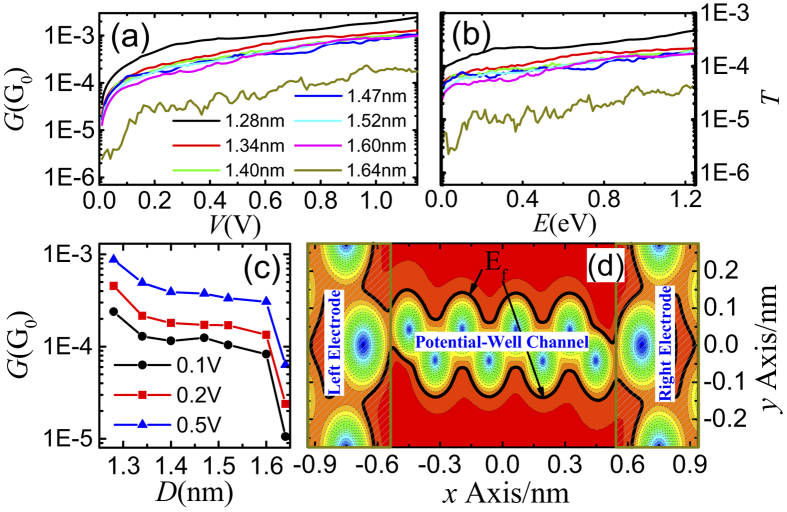
Steps in conductance trances. (**a**) Conductance as a function of bias voltage with different electrode distances for C6 molecular junction. (**b**) is the corresponding transmission probability curves. (**c**) Conductance as a function of electrode distance(*D*) with different bias voltages, (**d**) Spatial distribution of effective potentials on the plane across the centers of C and N atoms for C6 molecular junction shows a coherent potential-well channel is formed for the well connected molecular junction.

In order to understand the reason that conductance trances often show steps when stretching molecular junctions, we further investigate the conductance and transmission probability after calculating the numerical effective potential values with the electrodes in different distances for C6 molecular systems (Fig. 2(a,b)). The figure shows that, when the molecule is connected with two electrodes by two linker groups, not only the conductance values but also the variation trend of the *G*-*V* curves change only a little for the electrodes in different distances. However, when the molecular junction is ruptured at 1.64 nm, the conductance is distinctly smaller than those with shorter electrode distances, and the transmission probabilities show the similar character to the conductance. Figure 2(c) shows that, with elongation of the molecular junction, the conductance changes only a little until the molecular junction is ruptured, *i.e.*, the steps are emerged in the conductance traces, which can be understood from OTCTCA method. As mentioned above, the potential wells and potential barriers are the dominant factors that influence the electronic transport through molecular junctions. However, the numerical results show that, when the molecule is well linked with two electrodes, a potential-well channel which is well connected with the two electrodes is formed and no potential barrier cuts off the coherent potential-well channel (see Fig. 2(d)). Moreover, when elongating the molecular junction, the change of the potential-well channel has only a little influence on transmission probability. However, when the molecular junction is ruptured by further stretching, a potential barrier appears at the ruptured point, and the relaxation of the molecule and the electrodes makes the potential barrier broadening rapidly just after the molecular junction is broken. The transmission probabilities of electronic transporting through a potential barrier generally exponentially decay rapidly with the increase of the width and height of the potential barrier. That is to say, the transmission probability is very sensitive to the width and height of potential barrier, but not sensitive to that of potential well. Thus, one can easily understand why steps often present in the conductance traces when stretching molecular junctions in experiment.

### Double conductance steps

**Figure 3:**
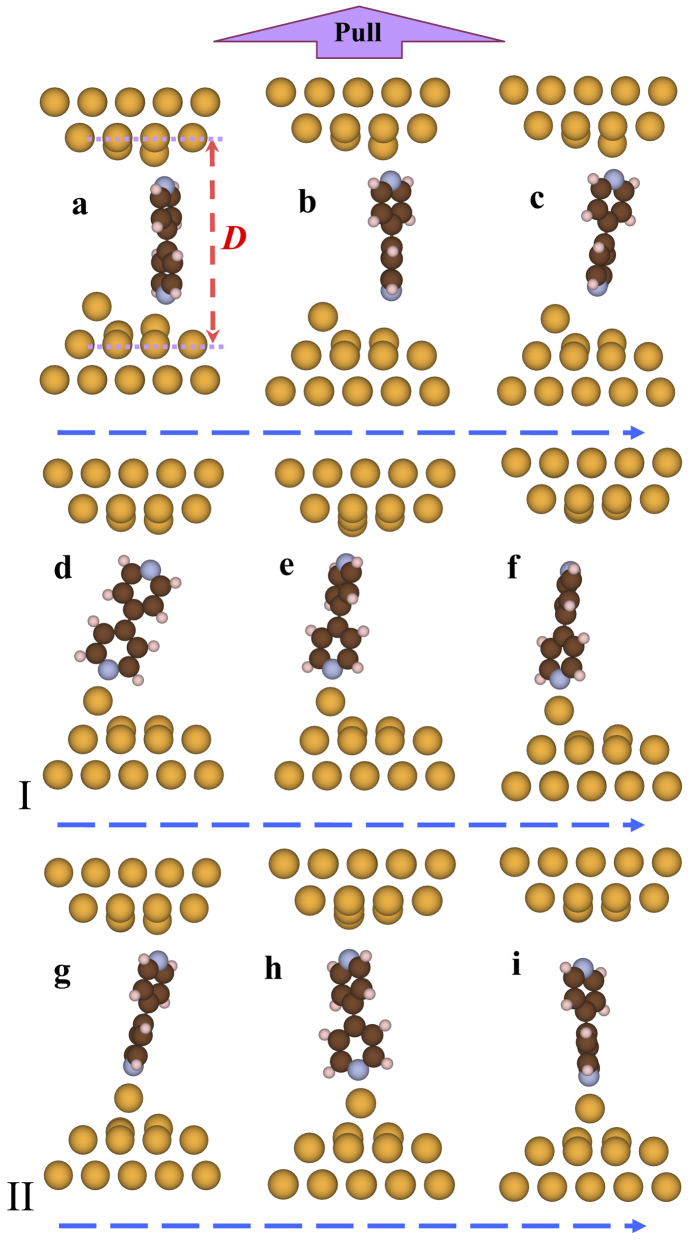
Elongation process of 4, 4′-bipyridine molecular junction. Two probable processes for elongation of 4, 4′-bipyridine molecular junction with one electrode possessing a surface Au atom, which are denoted as process I (c → d → f) and process II(c → g → i).

4, 4′-bipyridine molecular junction is also investigated by OTCTCA method. In order to simulate the stretching process, considering existence of single surface Au atom on the tip of breakpoint, we construct a 4, 4′-bipyridine molecular junction as Fig. 3 shows. At first, the 4, 4′-bipyridine molecule is more than 0.4 nm far away from the surface Au atom. The stretching process is simulated at B3LYP level with Lanl2DZ basis set in Gaussian03 packages^52^. In the geometric optimization, 4, 4′-bipyridine molecule and the Au atoms close to the molecule are relaxed as the electrode distance being elongated gradually. The numerical results show that, when the electrode distance is shorter than 1.53 nm (Fig. 3a,b), the molecule links with electrodes on the flat part of the gold tips. When the electrode distance is elongated to about 1.53 nm, if the molecule is exactly broken from the electrode with single surface Au atom on the gold tip and close to the end of the molecule, two interesting processes will probably take place. One is that the end of the molecule straight shifts to adsorb on the surface Au atom, and further elongation of the electrodes induces the other end of the molecule gliding on the other gold tip until the molecule is approximately perpendicular to the surface of gold tip. Finally, at 1.59 nm, the molecule is broken down from the gold tip without surface Au atom (see process I of Fig. 3, *i.e.*, c → d → e → f). The second probable process is that, the end of the molecule shifts to close the surface Au atom, simultaneously, the surface Au atom glides on the gold tip from one hollow position to the neighbor hollow position to close the end of the molecule, and further elongation also induces the other end of the molecule gliding on the other gold tip until the molecule is approximately perpendicular to the surface of gold tip. Finally, at 1.63 nm, the molecule is broken down from the gold tip without surface Au atom (see process II of Fig. 3, *i.e.*, c → g → h → i). However, if the molecule is broken from the electrode without surface Au atom on the tip, the molecular junction is straight ruptured.

**Figure 4:**
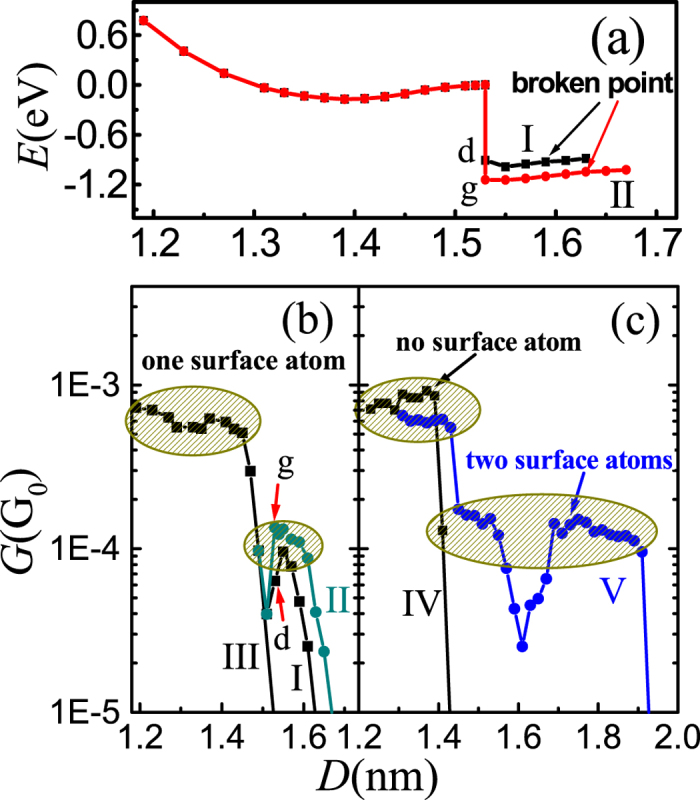
Energy and conductance traces of 4, 4′-bipyridine molecular junction. (**a**) Variation of single-point energy in the stretching process of the molecular junction, (**b**,**c**) Conductance traces in the stretching process of the molecular junction with 25mV bias voltage.

In order to understand why the end of 4, 4′-bipyridine molecule is prone to adsorbing on the surface Au atom, we show the evolution of single-point energy when elongate 4, 4′-bipyridine molecular junction in Fig. 4(a). From the figure one can see that, when the molecule turns to adsorbing on the surface Au atom at about 1.53 nm, the single-point energy is sharply dropped about 1.0 eV. This result implies that, when the electrodes are elongated to a certain distance, the neighboring surface Au atom exhibits strong attractive force to the end of 4, 4′-bipyridine molecule to prevent the molecule from straight breaking at the flat part of the gold tip, although at this distance the surface Au atom is about 0.5 nm far away from 4, 4′-bipyridine molecule. Additionally, Fig. 4(a) also shows that, when elongating the molecular junction, the molecule is very easy to glide on the gold surface as well as the surface Au atom.

Figure 4(b,c) show the conductance traces of stretching process of 4, 4′-bipyridine molecular junction, where (b) is for the molecular junction just as Fig. 3 shows, while (c)-IV is for the molecular junction without surface Au atom on the tips of gold electrodes and (c)-V is for the junction with two electrodes both possessing single surface Au atom. From Fig. 4(b,c), one can see that, in the stretching process, all of the conductance traces show high steps no matter whether there exists surface Au atom or not. If there is no surface Au atom on the gold tips, the conductance decreases straightly when the molecule is broken down from either electrode tip. However, if there is a surface Au atom on one electrode tip and the molecule turns to link with the surface Au atom as processes I and II shown in Fig. 3, a second step in the conductance traces is often appeared (Curve I and II in Fig. 4(b)), although there exist very small conductance values at about 1.51 nm. These small conductance values are due to the critical state that the molecule is about broken from the flat part of the gold tip and before it links with the surface Au atom. Actually, this critical state is generally unstable due to the thermal vibration of the molecular systems. Thus, one can expect that this very low conductive state is uneasy to probe in the experiment. The second step is always lower than the first step, where the conductance value of the second one is about 1/6 to 1/4 of the first one, which agrees with experiment probes of Quek *et al.* very well^49^,^50^,^51^. Additionally, the width of the lower steps is obviously narrower than that of the higher steps, which attributes to the situation that the easier absorption of molecule on the surface Au atom would induce the easier breaking of molecule from the other flat gold tip. The width of the step for process II is about 0.12 nm, but it is very narrow for process I, which seems to indicate that the width of the lower step is uncertain which consists with the uncertainty of the breaking process of the stretched molecular junction. The experimental probes also showed that the lower steps are very narrow and with uncertain width^49^,^50^. If the molecule is broken from the electrode without surface Au atom on the tip, the conductance is found to decrease straightly at the broken distance (Curve III in Fig. 4(b)). The numerical results also show that there is some probability for the molecule broken from the flat part straightly although there is a surface Au atom on the tip. Thus, one can understand why only a small proportion for the molecular junction shows lower conductive state in experiment^49^. Additionally, the distance between the surface Au atom and the neighbor Au layer is about 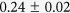 nm, which exactly corresponds to the distance for switching the molecular junction between higher and lower conductive states in the experiment probing^49^. Furthermore, due to the surface Au atom, the distance to push-back the two electrodes to contact with each other from low conductive state is estimated to be about 1.0~1.1 nm, which is also consistent with Quek’s probes very well^49^.

When the two electrodes both possess single surface Au atom, the conductance trace still shows two conductance steps (Fig. 4 (c)). From Fig. 4(c), one can see that there is no very lower conductance value during the conductance trace dropping from the high step to the low step, which is different from the case of only one electrode possessing single surface Au atom. The reason is that the end of the molecule is easier to adsorb on the surface Au atom because of the existence of the surface Au atom at the other electrode. It is noticeable that, if the two surface Au atoms do not straight face to each other, a valley in the middle part of low conductance step appears which also attributes to the critical state before the molecule adsorbs on the other surface Au atom. However, the thermal vibration of the molecular system may also reduce or eliminate this valley. Nevertheless, the appearance of this wider low conductance step in experimental probing has little probability because the configuration is seldom formed.

## Discussion

It is not an occasional event to successfully simulate the dependence of conductance on molecular length and the conductance steps in the stretching processes of the molecular junctions with OTCTCA method. In fact, these results are inevitable due to the wave character of electrons. As one knows, for the electronic transport in the molecular junction, the electronic waves are injected into the functional molecule from electrode and forced to transfer along the negative bias direction in the molecule by external bias voltage. Simultaneously, the atoms in the molecule can be considered as barriers or scattering centers in the path of transmission waves, which result in the diffracting and reflecting of electronic waves. One can find that the OTCTCA method exactly matches with this physical picture. Additionally, the OTCTCA calculations show that the shape of the effective potential and the number of scattering centers are the key factors to influence nonresonant transmission, which not only matches the essential characteristic of nonresonant transmission, but also matches the original predicted mechanism of exponential length dependence of the conductance^56^,^57^. It is well known that the non-equilibrium Green’s function method is a more sophisticated theory to study electronic transport of molecular junction, which is very helpful for understanding the conductive mechanisms of molecular device^11^. However, for nonresonant transmission, the OTCTCA method seems possessing three advantages, *i.e.*, inexpensive computational cost, intelligible electronic transport picture, and well consistent results. The disadvantage of OTCTCA method is difficult to deal with longer molecular chain with large bending angle. For the strong coupling molecular junctions such as molecular device with dithiol linker groups, the strong coupling may enhance the conductivity of the molecular junction for some extent, which is not included in the present OTCTCA method and will be considered in future.

In summary, based on hybrid density functional theory, a method of one-dimension transmission combined with three-dimension correction approximation is developed and the electron-transport properties of alkane diamines and 4, 4′-bipyridine molecular junctions are investigated. The conductance of alkane diamines decays exponentially with the increase of CH_2_ unit in the alkane diamines molecular chains with decay constant of about 0.85~0.95 per CH_2_ unit. Conductance trances are calculated by elongating molecular junctions successively and steps are found in the conductance traces. Especially, for 4, 4′-bipyridine molecular junction, the conductance trances show bi-stable conductive states as the experimental probes, which is induced by single surface Au atom that lies on the tip of gold electrode. The exponential decaying of length dependence and the existence of conductance steps both indicate that, in the lower bias regime, nonresonant electronic transmission dominates electronic transport of molecular device.

## Methods

A molecular device consists of a functional molecule sandwiched between two metal electrodes. In electronic transport process, by driving of bias voltage, the electrons are injected into functional molecule from one electrode (defined as source reservoir) and scattered by the molecule, at last poured into the other electrode (defined as drain reservoir). It is obvious that the spatial distribution of the effective potential that around the molecule dominates electrons transmit through the molecule. The transmission probability in the direction of bias voltage (defined as positive *x* axis) and the state density of reservoirs are two key factors when calculate current and conductance of molecular device.

If the incident probability current density is *j*_*x*_, the transmission probability through the molecule is *T*(*E*), then the current that flows through molecular device is


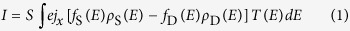


where 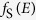 and 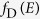 are the Fermi distribution functions of source and drain reservoirs, 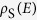 and 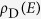 are the densities of energy states per unit volume of reservoirs. *S* is the effective inject area. Here we apply plane wave to approximately simulate incident wave, thus the probability current density^58^


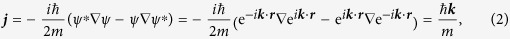


and 
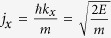
, where *E* is the energy of incidence electron relative to the Fermi energy of drain reservoir.

**Figure 5:**
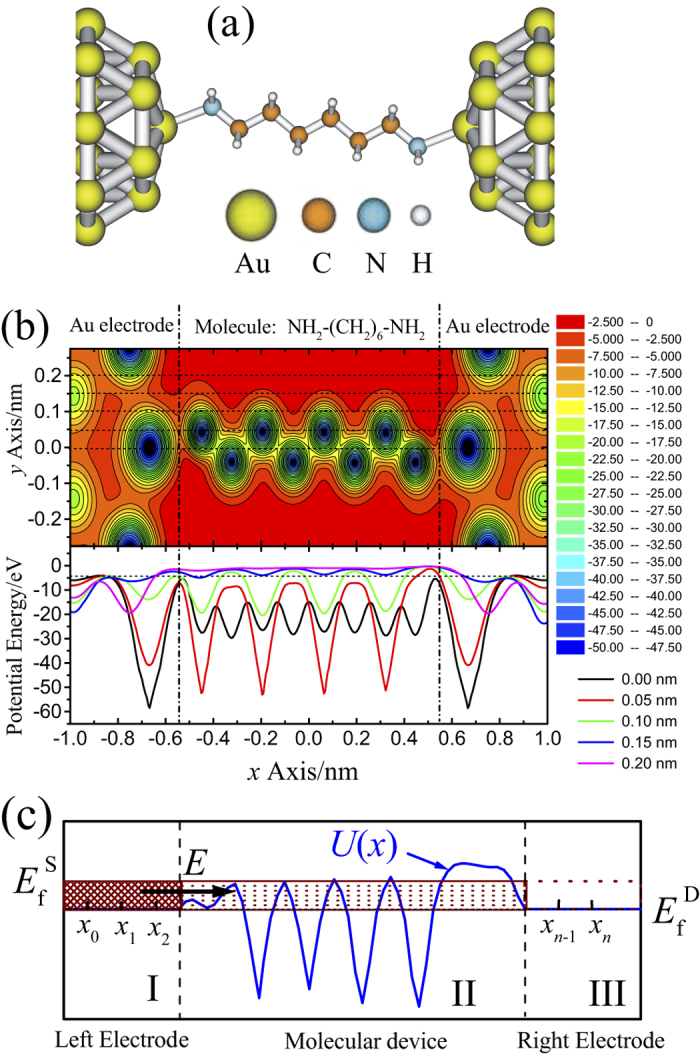
Effective potential distribution and one-dimension electron transmission scheme. (**a**) 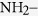

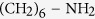
 molecular junction. (**b**) Spatial distribution of effective potentials on the plane across the centers of C and N atoms. The lower part of (**b**) shows the potential distributions along the dashed lines that the upper part of (**b**) shows. (**c**) Scheme of electrons transmitting through one-dimension potential field.

In order to obtain transmission probability *T*(*E*), we plot spatial distribution of effective potential of a molecule in Fig. 5(b) which is calculated at B3LYP level with Lanl2DZ basis set in Gaussian03 packages^52^. From the figure, one can see that, for the incident electrons, the molecule offers a space of effective potential field which consists of potential well and potential barrier. Thus, just as one-dimension transmission, for a given energy, the electrons must be with a specific probability to transmit from one electrode to the other electrode which relates to the spatial distribution of a three-dimension potential field around functional molecule. Considering that only *x* components of probability current density have contribution to the current, we developed one-dimension transmission combined with three-dimension correction method to approximately calculated transmission probability of molecular device. In this method, we calculate a series of transmission probability *τ*_*i*_s of electrons with given energy along a corresponding series lines that parallel *x* axis (as dashed line in upper part of Fig. 5(b)) with spatial distribution of effective potential 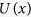 at first. Then we average the series *τ*_*i*_s as the approximate statistic transmission probability of the molecule, which is written as 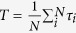. Generally, about 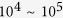 *τ*_*i*_s will be computed to obtain average transmission probability *T*, and all of the *τ*_*i*_s are carried out numerically from Schrödinger equation.

From lower part of Fig. 5(b) one can see that the effective potential distribution 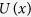 along each line forms irregular one-dimension potential wells and/or potential barriers for electronic transmission. Suppose the incident energy of electrons is *E*, then the time-independent Schrödinger equation can be written as


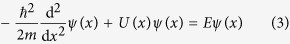


Since the effective potential distribution 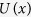 is irregular and can’t be expressed by a simple function, the solution of Schrödinger equation can only be obtained numerically by differential method. According to the structure of molecular junction, we divide the space into three areas, left electrode (area I), molecule/scattering area (area II) and right electrode (area III). For the electrodes of area I and area III, we apply plane wave approximation and neglect the influence of potential fluctuate on the wave functions, suppose the electrons are injected from area I, then the wave function in area I can be written as^58^


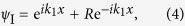


which consists of incident wave and reflected wave. The wave function in area III only consists of transmission wave which is written as


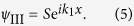


In area II, the wave function is as


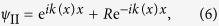


where 
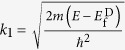
, 
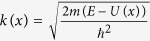
 and 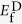 is the Fermi energy of drain electrode. In order to calculate transmission coefficient *S*, we divide transmission area *x*_0_ to *x*_*n*_ into *n* equal segments, then the ratio of wave functions of neighbor points is 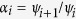, and applying 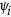, we have


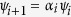


and


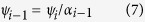



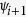
 and 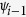 can be expressed by Taylor series as


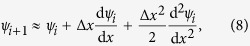



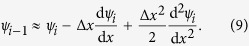


From equation (8) and (9), we have


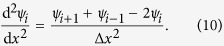


From equation (10) and with the help of Schrödinger equation (3), we can obtain


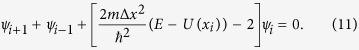


Let 
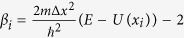
, from equation (7) and (11), we can deduce the recurrence relations between wave ratios as


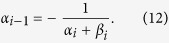


Additionally, 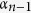 can be derived from area III as


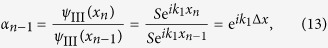


and with the help of recurrence relations we further derive 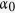 from 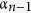. Moreover, from area I, 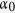 can be written as


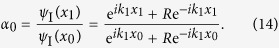


Then we obtain the reflected coefficient


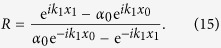


Based on the particle conservation law we know that 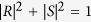, thus we obtain the transmission probability


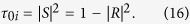


However, considering three-dimension effect, especially the diffraction effect of wave function in three-dimension space, the transmission probability obtained by one-dimension approximation is generally much larger than the exact value. In fact, the molecule consists of series scattering centers, *i.e.*, the atoms, thus for lower incident energy, only the *s* partial wave has obvious contribution to the scattering. Considering that *s* partial wave is isotropic, one can easily understand that, when electrons pass through each scattering center, only a half of electrons are scattered forward. Hence the transmission probability should be reduced by half when the electrons pass each scattering center, *i.e.*, 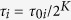, where *K* is the number of scattering centers in the transmission path. In addition, the coupling between different lines will cause the electrons pass through more scattering centers compared with those only transporting along the line parallel *x* axis, which will further reduce the transmission probability to about 0.9 ± 0.1, and the exact proportion of reducing is related to the geometries of molecules.

## Additional Information

**How to cite this article**: Liu, R. *et al.* A method to study electronic transport properties of molecular junction: one-dimension transmission combined with three-dimension correction approximation (OTCTCA). *Sci. Rep.* **6**, 21946; doi: 10.1038/srep21946 (2016).

## Supplementary Material

Supplementary Information

## References

[b1] Reed M. A., Zhou C., Muller C. J., Burgin T. P. & Tour J. M. Conductance of a molecular junction. Science 278, 252–254 (1997).

[b2] Sun M., Zhang Z., Zheng H. & Xu H. In-situ plasmon-driven chemical reactions revealed by high vacuum tip-enhanced Raman spectroscopy. Sci. Rep. 2, 647 (2012). 22970339 10.1038/srep00647 PMC3438462

[b3] Cui X. D. *et al.* Reproducible measurement of single-molecule conductivity. Science 294, 571–574 (2001). 11641492 10.1126/science.1064354

[b4] Xu B. & Tao N. J. Measurement of single-molecule resistance by repeated formation of molecular junctions. Science 301, 1221–1223 (2003). 12947193 10.1126/science.1087481

[b5] Cun H. Y. *et al.* Tuning structural and mechanical properties of two-dimensional molecular crystals: the roles of carbon side chains. Nano Lett. 12, 1229–1234 (2012). 22375560 10.1021/nl203591t

[b6] Sun M., Zhang Z., Chen L. & Xu H. Tip-enhanced resonance couplings revealed by high vacuum tip-enhanced raman spectroscopy. Adv. Opt. Mater. 25, 449–455 (2013).

[b7] Nazin G. V., Qiu X. H. & Ho W. Visualization and spectroscopy of a metal-molecule-metal bridge. Science 302, 77–81 (2003). 12958368 10.1126/science.1088971

[b8] Xu B. Q., Li X. L., Xiao X. Y., Sakaguchi H. & Tao N. J. Electromechanical and conductance switching properties of single oligothiophene molecules. Nano Lett. 5, 1491–1495 (2005). 16178263 10.1021/nl050860j

[b9] Capozzi B. *et al.* Length-dependent conductance of oligothiophenes. J. Am. Chem. Soc. 136, 10486–10492 (2014). 25003761 10.1021/ja505277z

[b10] Li Z. L., Zou B., Wang C. K. & Luo Y. Electronic transport properties of molecular bipyridine junctions: effects of isomer and contact structures. Phys. Rev. B 73, 075326 (2006).

[b11] Brandbyge M., Mozos J. L., Ordejón P., Taylor J. & Stokbro. K. Density-functional method for nonequilibrium electron transport. Phys. Rev. B 65, 165401 (2002).

[b12] Jiang J., Kula M. & Luo Y. A Generalized quantum chemical approach for elastic and inelastic electron transports in molecular electronics devices. J. Chem. Phys. 124, 034708 (2006). 16438601 10.1063/1.2159490

[b13] Soler J. M. *et al.* The SIESTA method for *ab initio* order-N materials simulation. J. Phys.: Condens. Matter 14, 2745–2779 (2002).

[b14] Zelovich T., Kronik L. & Hod O. State representation approach for atomistic time-dependent transport calculations in molecular junctions. J. Chem. Theory Comput. 10, 2927–2941 (2014). 26588268 10.1021/ct500135e

[b15] Wang C. K. & Luo Y. Current–voltage characteristics of single molecular junction: dimensionality of metal contacts. J. Chem. Phys. 119, 4923–4928 (2003).

[b16] Li G. *et al.* Compensation of coulomb blocking and energy transfer in the current voltage characteristic of molecular conduction junctions. Nano Lett. 12, 2228–2232 (2012). 22463365 10.1021/nl204130d

[b17] Ben-Moshe V., Rai D., Skourtis S. S. & Nitzan A. Steady-state current transfer and scattering theory. J. Chem. Phys. 133, 054105 (2010). 20707524 10.1063/1.3466876

[b18] Maiti S. K. & Nitzan A. Mobility edge phenomenon in a hubbard chain: A mean field study. Phys. Lett. A 377, 1205–1209 (2013).

[b19] Li Y., Feng Y. & Sun M. Photoinduced charge transport in a BHJ solar cell controlled by an external electric field. Sci. Rep. 5, 13970 (2015). 26353997 10.1038/srep13970 PMC4564800

[b20] Araidai M. & Tsukada M. Theoretical calculations of electron transport in molecular junctions: inflection behavior in fowler-nordheim plot and its origin. Phys. Rev. B 81, 235114 (2010).

[b21] Wang C. K., Fu Y., & Luo Y. A quantum chemistry approach for current-voltage characterization of molecular junctions. Phys. Chem. Chem. Phys. 3, 5017–5023 (2001).

[b22] Ventra M. D. Pantelides S. T. & Lang N. D. First-principles calculation of transport properties of a molecular device. Phys. Rev. Lett. 84, 979–982 (2000). 11017420 10.1103/PhysRevLett.84.979

[b23] Prodan E. & Car R. Theory of tunneling transport in periodic chains. Phys. Rev. B 76, 035124 (2007).

[b24] Zhang G. P., Hu G. C., Song Y., Li Z. L. & Wang C. K. Modulation of rectification in diblock co-oligomer diodes by adjusting anchoring groups for both symmetric and asymmetric electrodes. J. Chem. Phys. C 116, 22009–22014 (2012).

[b25] Su W., Jiang J., Lu W. & Luo Y. First-principles study of electrochemical gate-controlled conductance in molecular junctions. Nano Lett. 6, 2091–2094 (2006). 16968031 10.1021/nl061376z

[b26] Venkataraman L. *et al.* Single-molecule circuits with well-defined molecular conductance. Nano Lett. 6, 458–462 (2006). 16522042 10.1021/nl052373+

[b27] Chen F., Li X., Hihath J., Huang Z. & Tao N. Effect of anchoring groups on single-molecule conductance: comparative study of thiol-, amine-, and carboxylic-acid-terminated molecules. J. Am. Chem. Soc. 128, 15874–15881 (2006). 17147400 10.1021/ja065864k

[b28] Huang M. J. *et al.* Conductance of tailored molecular segments: A rudimentary assessment by Landauer formulation. J. Am. Chem. Soc. 136, 1832–1841 (2014). 24437396 10.1021/ja4088538

[b29] Park Y. S. *et al.* Contact chemistry and single-molecule conductance: A comparison of phosphines, methyl sulfides, and amines. J. Am. Chem. Soc. 129, 15768–15769 (2007). 18052282 10.1021/ja0773857

[b30] Arroyo C. R. *et al.* Influence of binding groups on molecular junction formation. J. Am. Chem. Soc. 133, 14313–14319 (2011). 21806051 10.1021/ja201861k

[b31] Chen W. *et al.* Highly conducting π-conjugated molecular junctions covalently bonded to gold electrodes. J. Am. Chem. Soc. 133, 17160–17163 (2011). 21939263 10.1021/ja208020j

[b32] Kronemeijer A. J. *et al.* Universal scaling of the charge transport in large-area molecular junctions. Small 11, 1593–1598 (2011). 21538870 10.1002/smll.201100155

[b33] Hong W. *et al.* Single molecular conductance of tolanes: experimental and theoretical study on the junction evolution dependent on the anchoring group. J. Am. Chem. Soc. 134, 19425–19431 (2012). 22175273 10.1021/ja209844r

[b34] Jiang J., Lu W. & Luo Y. Length dependence of coherent electron transportation in metal–alkanedithiol–metal and metal–alkanemonothiol–metal junctions. Chem. Phys. Lett. 400, 336–340 (2004).

[b35] Paulsson M., Krag C., Frederiksen T. & Brandbyge M. Conductance of alkanedithiol single-molecule junctions: a molecular dynamics study. Nano Lett. 9, 117–121 (2009). 19061346 10.1021/nl802643h

[b36] Bagrets A., Arnold A. & Evers F. Conduction properties of bipyridinium-functionalized molecular wires. J. Am. Chem. Soc. 130, 9013–9018 (2008). 18570418 10.1021/ja800459k

[b37] Asai Y. & Fukuyama H. Theory of length-dependent conductance in one-dimensional chains. Phys. Rev. B 72, 085431 (2005).

[b38] Prodan E. & Car R. Tunneling conductance of amine-linked alkyl chains. Nano Lett. 8, 1771–1777 (2008). 18489169 10.1021/nl8012133

[b39] Yu J. X., Hou Z. W. & Liu X. Y. Stability of conductance oscillations in carbon atomic chains. Chin. Phys. B 24, 067307 (2015).

[b40] Fu X. X., Zhang R. Q., Zhang G. P. & Li Z. L. Rectifying properties of oligo(phenylene ethynylene) heterometallic molecular junctions: molecular length and side group effects. Sci. Ref. 4, 06357 (2014). 10.1038/srep06357 PMC4163668 25220880

[b41] Basch H., Cohen R. & Ratner M. A. Interface geometry and molecular junction conductance: geometric fluctuation and stochastic switching. Nato Lett. 5, 1668–1675 (2005). 10.1021/nl050702s 16159203

[b42] Bao D. L. *et al.* Theoretical study on mechanical and electron-transport properties of conjugated molecular junctions with carboxylic or methyl sulfide links. Phys. Lett. A 378, 1290–1295 (2014).

[b43] Liu R. *et al.* Study on force sensitivity of electronic transport properties of 1,4-butanedithiol molecular device. Acta Phys. Sin. 63, 068501 (2014).

[b44] Moreno-García P. *et al.* Single-molecule conductance of functionalized oligoynes: length dependence and junction evolution. J. Am. Chem. Soc. 135, 12228–12240 (2013). 23875671 10.1021/ja4015293

[b45] Tang Y. H., Bagci V. M. K., Chen J. H. & Kaun C. C. Conductance of stretching oligothiophene single-molecule junctions: a first-principles study. J. Phys. Chem. C 115, 25105–25108 (2011).

[b46] Sun M., Zhang Z., Kim Z. H., Zheng H. & Xu H. Plasmonic scissors for molecular design. Chem. Eur. J. 19, 14958–14962 (2013). 24038434 10.1002/chem.201302610

[b47] Sun M., Fang Y., Zhang Z. & Xu H. Activated vibrational modes and Fermi resonance in tip-enhanced Raman spectroscopy. Phys. Rev. E 87, 020401 (2013). 10.1103/PhysRevE.87.020401 23496445

[b48] Choi H. J., Cohen M. L. & Louie S. G. First-principles scattering-state approach for nonlinear electrical transport in nanostructures. Phys. Rev. B 76, 155420 (2007).

[b49] Quek S. Y. *et al.* Mechanically controlled binary conductance switching of a single-molecule junction. Nat. Nanotechnol. 4, 230–234 (2009). 19350032 10.1038/nnano.2009.10

[b50] Kamenetska M. *et al.* Conductance and geometry of pyridine-linked single-molecule junctions. J. Am. Chem. Soc. 132, 6817–6821 (2010). 20423080 10.1021/ja1015348

[b51] Kim T. *et al.* Determination of energy level alignment and coupling strength in 4,4′-Bipyridine single-molecule junctions. Nano Lett. 14, 794–798 (2014). 24446585 10.1021/nl404143v

[b52] Frisch M. J. *et al.* Gaussian Inc. (2004). *Gaussian 03 Revision E.01*, Wallingford CT.

[b53] Frei M., Aradhya S. V., Koentopp M., Hybertsen M. S. & Venkataraman L. Mechanics and chemistry: single molecule bond rupture forces correlate with molecular backbone structure. Nano Lett. 11, 1518–1523 (2011). 21366230 10.1021/nl1042903

[b54] Hong W. *et al.* Single molecular conductance of tolanes: experimental and theoretical study on the junction evolution dependent on the anchoring group. J. Am. Chem. Soc. 134, 2292–2304 (2012). 22175273 10.1021/ja209844r

[b55] Perrin M. L. *et al.* Large negative differential conductance in single-molecule break junctions. Nat. Nanotechnol. 9, 830–834 (2014). 25173832 10.1038/nnano.2014.177

[b56] Wold D. J., Haag R. Rampi M. A. & Frisbie C. D. Distance dependence of electron tunneling through self-assembled monolayers measured by conducting probe atomic force microscopy: unsaturated versus saturated molecular junctions. J. Phys. Chem. B 106, 2813–2816 (2002).

[b57] Holmlin R. E. *et al.* Electron transport through thin organic films in metal-insulator-metal junctions based on self-assembled monolayers. J. Am. Chem. Soc. 123, 5075–5085 (2001). 11457338 10.1021/ja004055c

[b58] Shankar R. Principles of Quantum Mechanics 2nd edn, Ch. 5, 164–175 (New York, 1994).

