# Supplementary material for: A method to study electronic transport properties of molecular junction: one-dimension transmission combined with three-dimension correction approximation (OTCTCA)
Source: Sci Rep. 2016 Feb 25;6:21946. doi: 10.1038/srep21946 (PMC4766509; doi:10.1038/srep21946)
Supplement: Supplementary Information [file srep21946-s1.doc]

**A method to study electronic transport properties of molecular junction: one-dimension transmission combined with three-dimension correction approximation (OTCTCA)**

Ran Liu, Chuan-Kui Wang and Zong-Liang Li^*^

School of Physics and Electronics, Shandong Normal University, Jinan, 250014, China

^*^Correspondence and requests for materials should be addressed to Z. L. Li ([lizongliang@sdnu.edu.cn](mailto:lizongliang@sdnu.edu.cn))

**Supplementary Information**

**1. The stretching processes of alkane diamine molecular junctions of C2, C4, C6 and C8 are simulated.** In the simulating, we relaxed the atoms of the functional molecules and the Au atoms neighbor to functional molecule and fixed the other Au atoms to perform geometric optimization. Then adjust the fixed Au atoms to increase the electrode distance(*D*) and perform geometric optimization step by step. The initial geometry of each step is the optimized geometry of the former step except for the fixed Au atoms being adjusted a little. The ground state energies(*E*) of the molecular systems vs electrode distances are shown in Figure S1. Use $F=\partial E/\partial D$, we further calculated the force of electrodes on the molecules, which are also shown in Figure S1, where positive values corresponding to stretching forces and negative value corresponding to pressure.


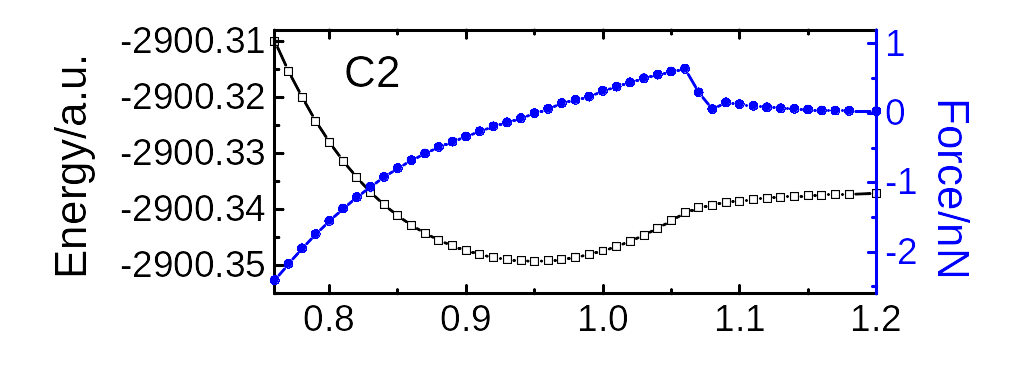

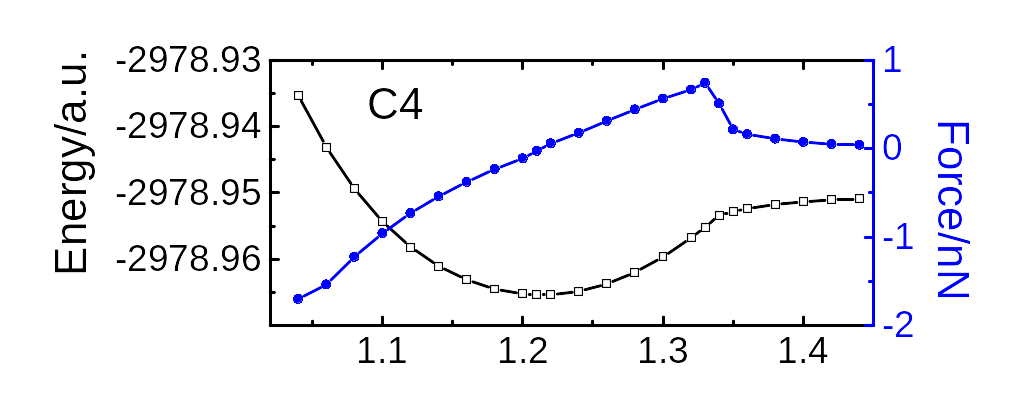

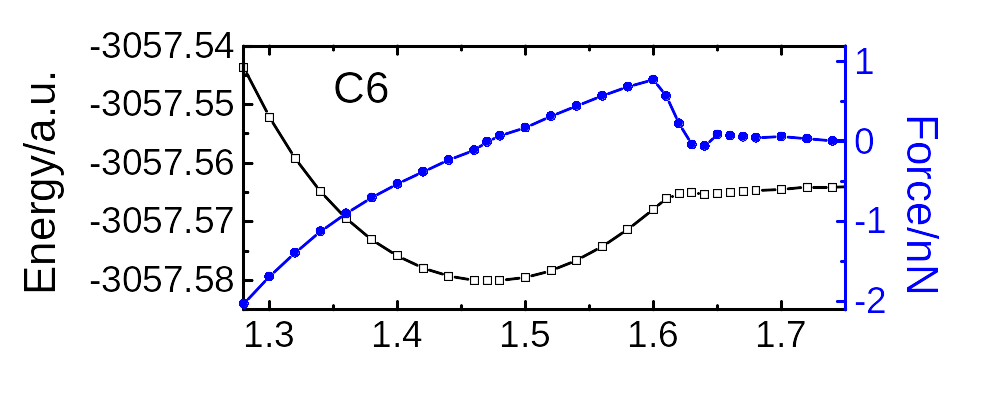

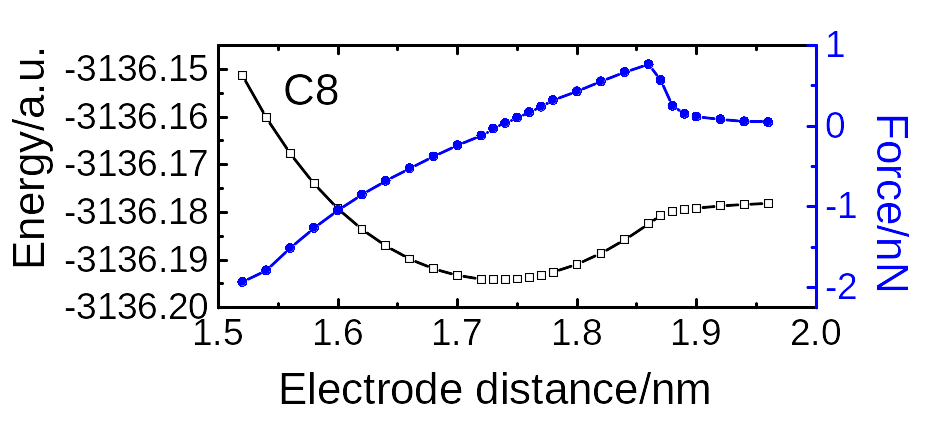


Figure S1. The ground state energies and the forces as functions of electrode distance for alkane diamine molecular junctions of C2, C4, C6 and C8.

2. **NEGF calculations for alkane diamine molecular junctions of C2, C4, C6 and C8**

The transmission spectra are calculated using density functional theory (DFT) and the non-equilibrium Green’s function formalism (NEGF) implemented in TranSIESTA module of SIESTA software. In calculation the improved Troullier-Martins type norm-conserving pseudopotentials are used to describe the core electrons and the Perdew-Burke-Ernzerhof (PBE) generalized gradient approximation (GGA) is adopted for the exchange-correlation functional. In the transmission calculation a single-ζ plus single polarization (SZP) basis set is employed for Au atoms. Figure S2 shows the transmission of C2, C4, C6, C8 molecular junctions with bias voltage V=0.0 and 0.2V..

Figure S2 Transmission of C2, C4, C6, C8 molecular junctions with bias voltage V=0.0 and 0.2V..
